# Acute kidney injury following radical cystectomy and urinary diversion: predictors and associated morbidity

**DOI:** 10.1590/S1677-5538.IBJU.2017.0283

**Published:** 2018

**Authors:** Yasser Osman, Ahmed M. Harraz, Samer El-Halwagy, Mahmoud Laymon, Ahmed Mosbah, Hassan Abol-Enein, Atalla A. Shaaban

**Affiliations:** 1 Urology and Nephrology Center, Mansoura University, Egypt

**Keywords:** Acute Kidney Injury, Cystectomy, Urinary Diversion

## Abstract

**Introduction:**

Acute kidney injury (AKI) after major surgeries is associated with significant morbidity and mortality. We aim to report incidence, predictors and associated comorbidities of AKI after radical cystectomy in a large cohort of patients.

**Materials and Methods:**

We conducted a retrospective analysis of 1000 patients who underwent open radical cystectomy in a tertiary referral center. Perioperative serum creatinine measurements were used to define AKI according to the RIFLE criteria (as Risk, Injury and Failure). The predictors of AKI after surgery were determined using univariate and multivariate analyses.

**Results:**

Out of 988 evaluable patients, AKI developed in 46 (4.7%). According to RIFLE criteria; AKI-Risk, AKI-Injury and AKI-Failure occurred in 26 (2.6%), 9 (0.9%) and 11 (1.1%) patients, respectively. Multivariate analysis showed that performing nephroureterectomy with cystectomy (Odds ratio [OR]: 4.3; 95% Confidence interval [CI]: 1.3-13.6; p=0.01) and the development of high grade complications (OR: 3.8; 95% CI 1.9-7.2; p<0.0001) were independently associated with AKI.

**Conclusions:**

AKI is a significant morbidity after radical cystectomy and the term should be included during routine cystectomy morbidity assessment.

## INTRODUCTION

Radical cystectomy and urinary diversion continue to be the basic modality for treatment of localized muscle invasive bladder cancer in both genders (1). Following surgery, a wide scale of diversion-related complications have been described and extensively analyzed including gastro-intestinal, urinary and renal function complications (2).

Acute kidney injury (AKI) is a devastating co-morbidity that is commonly encountered in critically ill patients and after major surgeries and is associated with severe morbidity and mortality (3). It has been shown that patients who partially recovered from an episode of AKI are at higher risk of long-term mortality (4) and those who completely recovered from an episode of AKI are more likely to develop incident chronic kidney disease (CKD) (5) or even end-stage renal disease (6, 7). The incidence and predictors of AKI after major urologic surgeries are poorly studied in the literature and limited mainly to cardiothoracic and orthopedic surgeries (8-10). This study was conducted to investigate the incidence and predictors of AKI in a large cohort of patients undergoing radical cystectomy and urinary diversion.

## MATERIALS AND METHODS

### Study Design

A retrospective cohort study was conducted in 1000 patients underwent radical cystectomy and urinary diversion at our tertiary referral center between January 2004 and September 2009. The study received our internal review board approval with informed consent waived because of the retrospective nature of the study. Patients with missed data about serum creatinine (SCr) measurements were excluded (n=12). All patients underwent open radical cystectomy with standard pelvic lymphadenectomy up to the level of the common iliac artery and followed by urinary diversion. During the postoperative period, all ureters were stented for 11-to-13 days after orthotopic bladder substitution and 9 days after ileal conduit urinary diversion. Patients were kept in the hospital until catheter free; 21 days and 11 days for orthoptic and ileal conduit diversions, respectively.

#### Data Collection and Measurements

Data were collected from a prospectively maintained electronic database at our institution. Demographics included age, gender, body mass index (BMI) with obese patients defined as BMI >30, and the presence of diabetes mellitus or hypertension. Patient's co-morbidities were assessed using age-adjusted Charlson Co-morbidity index (CCI) as previously described (11). The presence of CKD was diagnosed as proposed by the National Kidney Foundation by having estimated glomerular filtration rate (eGFR) <60mL/min/1.7m^2^ (12). Baseline GFR was estimated using the Chronic Kidney Disease Epidemiology Collaboration Equation (CKD-EPI) (13). In patients presented with oliguria or anuria, measurements were obtained after decompressing the pelvicalyceal system by percutaneous nephrostomy tube and SCr measures had stabilized.

Recorded laboratory values included hemoglobin (anemia was defined as <10gm/dL) and albumin (hypoalbuminemia was defined as serum albumin <3.5gm/dL). The rate of blood loss was described as hemoglobin deficit and was calculated by the difference between preoperative and the lowest value of postoperative hemoglobin. Operative data included performing nephroureterectomy with cystectomy or not and type of the urinary diversion. The operating time was not reported in all patients; therefore, this item was eliminated from the analysis.

Postoperative complications were classified and graded according to the proposed modification of the Dindo-Clavien system (14) and grade I and II were considered minor and grades III to IV were considered major.

#### Outcome Assessment

The primary outcome of the study is the development of AKI. Three SCr measurements were used to define AKI. Baseline SCr is the nearest value before or at time of surgery. During the postoperative period, the peak SCr elevations and SCr at discharge were recorded. Patients with persistent rise of SCr at time of discharge were included. Patients with temporary transient rise were excluded from the study. Based on these readings, AKI was defined according to the RIFLE criteria by a persistent (till time of discharge) increase of SCr measurements 1.5 times the baseline value (15). Acute kidney injury was further classified into AKI-Risk (SCr increases >1.5 times the base line value), AKI-Injury (SCr increased >2 times the baseline value) and AKI-Failure (SCr increased >3 times the baseline value). As the scope of this study was limited to the perioperative period, AKI categories Loss of function and End-stage renal disease were not evaluated.

### Statistical analysis

Continuous variables were described as mean±SD for parametrically-distributed variables and median (interquartile range [IQR]) for nonparametric variables and nominal variables as frequencies (percentages) in each category. Age and BMI were described as continuous and nominal variables. We determined the incidence of AKI after radical cystectomy and urinary diversion. Continuous variables were compared between the two groups by student t test and categorical variables by Chi-square test. Patient's demographics, operative and postoperative data were tested for association with the occurrence of AKI. Significant factors were entered into a binary logistic regression model to determine the independent factors associated with AKI. Further sub-analysis of the cohort was performed excluding patients underwent nephroureterectomy with cystectomy, to account for a more homogenous study population.

## RESULTS

### Patient's Characteristics

A total of 988 patients (82.1% males) were eligible for the perioperative assessment of AKI. Of our study population, 72.9% were considered to have normal preoperative renal function (eGFR ≥ 60mL/min./m^2^). Ileal orthotopic bladder substitution was the most popular type of diversion and urothelial carcinoma was the most common histopathological type. Patient's demographics are displayed in Table-1.

**Table 1:** Demographics for patients undergoing radical cystectomy and urinary diversion.

		No. (988)
**Age, yr, mean, (SD)**		58 (8.2)
**Gender, no. (%)**		
	Male	811 (82.1)
	Female	177 (17.9)
**Urinary diversion, no. (%)**		
	Orthotopic	574 (58.1)
	Ileal conduit	387 (39.2)
	Continent cutaneous/rectal	27 (2.7)
**Histopathology, no. (%)**		
	TCC	690 (69.8)
	SCC	193 (19.5)
	Adenocarcinoma	61 (6.2)
	Others	44 (4.5)
**Tumor stage, no. (%)**		
	T_1_ or less	139 (14.1)
	T_2_	558 (56.5)
	T_3_	171 (17.3)
	T_4_	77 (7.8)
	T_x_	43 (4.4)
**N stage**		
	N_o_	676 (68.4)
	N_1_	106 (10.7)
	N_2_	130 (13.2)
	N_x_	76 (7.7)

**SD** = Standard deviation; **CKD** = Chronic kidney disease; **TCC** = Transitional cell carcinoma; **SCC** = Squamous cell carcinoma

### Incidence and independent variables associated with AKI after radical cystectomy

Acute kidney injury developed in 46 (4.7%) patients after radical cystectomy. According to RIFLE criteria; AKI-Risk, AKI-Injury and AKI-Failure occurred in 26 (2.6%), 9 (0.9%) and 11 (1.1%) patients, respectively.

Table-2 presents the association between the development of AKI and various study population characteristics. Comparing patients with and without AKI, there was no significant difference regarding age, gender, presence of DM, CKD or hypertension, BMI or preoperative SCr measurements. Similarly, type of urinary diversion did not attain significant association with the development of AKI after surgery.

**Table 2:** Demographics for patients with and without Acute Kidney Injury after Radical cystectomy.

		AKI, no. (%)		p-value
		No	Yes	
**Scale variables**				
**Age**, yr, mean (SD)		57.9 (8.3)	60 (7.1)	0.1
**Age adjusted CCI, mean (SD)**		**2.4 (1)**	**2.8 (0.9)**	**0.017**
**SCr, basal**, mg/dL, mean (SD)		1.3 (0.5)	0.9 (0.2)	0.1
**BMI**, mean (SD)		27.4 (4.9)	27.1 (4.7)	0.7
**HB**, deficit, gm/dL, median (IQR)		3.3 (2.5)	3.3 (2.3)	0.4
**Albumin**, gm/dL, mean (SD)		3.5 (0.4)	3.4 (0.4)	0.3
**Nominal variables**				
**Gender**				0.5
	Male	775 (95.6)	36 (4.4)	
	Female	167 (94.4)	10 (5.6)	
**DM**				0.4
	No	798 (95.6)	37 (4.4)	
	Yes	144 (94.1)	9 (5.9)	
**Hypertension**				0.3
	No	811 (95.6)	37 (4.4)	
	Yes	131 (93.6)	9 (6.4)	
**Hypoalbuminemia**				
	No	538 (95.9)	23 (4.1)	0.3
	Yes	404 (94.6)	23 (5.4)	
**Anemia**				0.7
	No	889 (95.3)	44 (4.7)	
	Yes	53 (96.4)	2 (3.6)	
**CKD**				0.1
	No	680 (94.4)	40 (5.6)	
	Yes	262 (97.8)	6 (2.2)	
**Obesity**				0.8
	No	668 (95.4)	32 (4.6)	
	Yes	274 (95.1)	14 (4.9)	
**Hydronephrosis**				0.6
	No	644 (95.1)	33 (4.9)	
	Yes	298 (95.8)	13 (4.2)	
**Nephroureterectomy with cystectomy**				**0.014**
	No	918 (95.6)	42 (4.4)	
	Yes	24 (85.7)	4 (14.3)	
**Type of diversion**				0.3
	Orthotopic	543 (94.6)	31 (5.4)	
	Loop	372 (96.1)	15 (3.9)	
	Others	27 (100)	0	
**Postoperative high grade complications**				< **0.0001**
	No	**818 (96.7)**	**28 (3.3)**	
	Yes	**124 (87.3)**	**18 (12.7)**	
Tumor stage				0.1
	Organ confined	701 (94.7)	39 (5.3)	
	Extravesical	241 (97.2)	7 (2.8)	

**CCI** = Charlson Comorbidity index; **SCr** = serum creatinine; **BMI** = body mass index; **HB** = hemoglobin; **DM** = Diabetes mellitus; **CKD** = Chronic kidney disease * analysis excluded patients with Tx

The mean±SD age-adjusted CCI was 2.8±0.9 for patients developing AKI after cystectomy vs. 2.4±1 for patients without AKI, a difference with statistical significance (p=0.017). Furthermore, AKI developed in 14.3% of patients undergoing nephroureterectomy at time of radical cystectomy vs. 4.4% in patients undergoing radical cystectomy without nephroureterectomy (p=0.014). Similarly, the development of high grade postoperative complications was significantly associated with the development of AKI after radical cystectomy (12.2% vs. 3.4% in patients with and without high grade complication; p<0.0001). The relationship between different grades of complications and the subcategories of AKI are shown in Table-3.

**Table 3:** Association between postoperative complications and the various stages of acute kidney injury after radical cystectomy.

Postoperative Complications*		Acute kidney injury classification, no. (%)			p-value
		Risk	Injury	Failure	
**I-II**					0.5
	**No**	20 (2.4)	8 (0.9)	10(1.2)	
	**Yes**	6 (4.4)	1 (0.7)	1 (0.7)	
**III**					**<0.001**
	**No**	21 (2.4)	8 (0.9)	3 (0.3)	
	**Yes**	5 (4.6)	1 (0.9)	8 (7.4)	
**IV**					**<0.001**
	**No**	24 (2.6)	8 (0.9)	4 (0.4)	
	**Yes**	2 (4.2)	1 (2.1)	7 (14.6)	

* Complications are cathegorized according to the modified Clavien system: **I, II** = minor; **III** = required intervention either by regional or general anestheisa; **IV** = single or multiorgan failure requiring intensive care unit admission

The three significant variables were entered into multivariate binary logistic regression analysis for determining independent variants associated with the development of AKI after radical cystectomy (Table-4). Only two variables remained statistically significant; performing nephroureterectomy with radical cystectomy had 4.3 times risk of development of AKI (p=0.01) and the development of high grade postoperative complications had 3.8 times risk of the development of AKI (p<0.0001). Furthermore, sub-analysis of the cystectomy cohort performed without nephroureterectomy during surgery, yielded orthotopic urinary diversion (Odds ratio (OR): 2.5; 95% Confidence interval (CI): 1.1-4.9; p=0.018), in addition to the development of high-grade postoperative complications, was significantly associated with 2.5 times rise in the likelihood of the development of AKI.

**Table 4:** Univariate and Multivariate Analyses for factors predicting Acute Kidney Injury after Radical cystectomy.

	Univariate analysis			Multivariate analysis		
	OR	95%CI	P-value	OR	95%CI	P-value
**Age-CCI**	1.424	1.065-1.905	0.017	1.324	0.985-1.778	0.061
**NU**	3.643	1.209-10.974	0.022	4.364	1.395-13.658	**0.011**
**High grade complications**	3.940	2.102-7.384	< 0.0001	3.802	1.996-7.242	**< 0.0001**

**CCI** = Charlson comorbidity index; **NU** = nephroureterectomy with cystectomy; **OR** = odds ratio; **CI** = Confidence interval

## DISCUSSION

Acute kidney injury is a well-studied co-morbidity after major surgical trauma notably cardiac surgeries (8, 9, 16). Radical cystectomy is a major surgical trauma by definition and urinary diversion is a well-known risk factor for renal function deterioration especially with ureteroileal anastomosis (UIA) obstruction (17). Therefore, incorporation of urinary diversion after radical cystectomy gives a special importance for studying AKI because of the increased burden on kidneys in such patients.

Early renal function changes after radical cystectomy were described in many reports in terms of acute renal failure. Schiavina et al. reported acute renal failure in 18 (4.4%) of their patients (18). Takada et al., in a multi-institutional study from Japan, reported an incidence of 0.5% of renal failure (19). Yuh and associates reported an overall incidence of 7.1% (14 out of 196 patients) that developed renal failure after robot-assisted radical cystectomy. The authors subcategorized the renal failure into 12 and 2 patients with minor and major complications, respectively (20). In the previous studies, the definition of postoperative renal failure is not clear, highlighting the lack in reporting AKI in the literature after radical cystectomy.

In this study, 46 (4.7%) of our patients developed persistent AKI at time of discharge. This incidence is relatively lower than reported after other major surgeries. After cardiac surgery, the incidence ranged from 8.9% up to 42% (8, 21) while after orthopedic surgery it was 16.8% (10). The probable higher risk of bleeding with its subsequent impact is anticipated in both cardiac and orthopedic surgeries. The use of cardiopulmonary bypass technique is a possible added factor in cardiac patients.

Many studies have described the association between various preoperative predictors and the occurrence of AKI after major surgeries (8, 16, 22). It has been shown previously that low eGFR is a significant predictor of AKI after major surgery (10, 22). However, this cannot be proved in our report probably because of the follow-up was limited to the peri-operative period. It has been shown that patients who experienced AKI were significantly associated with the development of later CKD on the long-term. Jones et al. showed that after a median follow-up of 2.5 years, 15% of patients with AKI, who were completely recovered at time of discharge, developed CKD with 5.9 folds increase in risk than those without AKI (5). Coca and associates in their systematic review about the incidence of CKD after AKI reported that patients with AKI had 8.8 and 3.1 folds increased risk for the development of CKD and ESRD, respectively (23). Therefore, the impact of AKI on the development of CKD on the long-term in this patient population is awaited.

Age-adjusted CCI is a cumulative score of multiple co-morbidities including diabetes mellitus, hypertension, cardiac and renal problems. These factors are known to affect renal function and therefore, patients with higher age-adjusted CCI are more likely to develop AKI in context of major surgical trauma as radical cystectomy. Nevertheless, our data failed to provide such evidence on multivariate analysis as an independent predictor. Generally, CCI is accepted as a mortality rather than a morbidity predictor (24). Although obesity was also shown to be associated with the development of AKI via oxidative stress mechanisms (25), BMI failed to predict AKI occurrence after radical cystectomy in this series probably because patients with morbid obesity are referred to radiotherapy as the primary modality of treatment in our practice.

In this contribution, performing nephroureterectomy with cystectomy was identified as a risk factor for the development of AKI irrespective of the type of urinary diversion. Probable explanations for these patients include possible prolongation of the operative time with subsequent increase in blood loss, hypotension and hypothermia as well as loss of functioning renal mass in patients performing nephrectomy for associated upper urothelial malignancy. Novara et al. reported significantly poor cancer-specific survival in patients with combined radical cystectomy and nephroureterectomy compared with isolated nephroureterectomy based on multi-institutional study (26). Conversely, in attempt to homogenize the cohort, we performed a sub-analysis excluding patients underwent nephroureterectomy with cystectomy, in this context, orthotopic substitution came into focus. It has been reported that the link between orthotopic substitution and renal function deterioration was attributed to the development of UIA stricture and to a less extent, pyelonephritis (27). Recently, there has been a focus on the effect of uri-nary diversion type on renal function. Gondo et al. has found that the type of urinary diversion was significantly correlated with renal function three months after surgery (28). Although this correlation was not maintained on multivariate analysis, this particular study is underpowered as smaller number of patients were included (164 patients). On the other hand, in a propensity matched analysis, the type of urinary diversion did not show an independent association with the development of CKD after surgery (29). Likewise, in patients with preoperative impaired renal function, continent diversion did not confer a risk for further renal function deterioration (30). It has to be noted that these two reports evaluated the long-term renal functional outcomes, and neither any of both had evaluated the development of AKI in the early postoperative period and its long-term effects. Therefore, a more in-depth look is essential to delineate this issue.

Higher grade complications were proved to be independent predictors for the development of AKI. Whether these complications are the cause or an effect of AKI is debatable and further analysis of the timing of the occurrence of AKI would provide us with more precise conclusion. Finally, the significant association between AKI and early post-operative mortality is one of the most important data derived from this work.

To the best of our knowledge, this work is the first to categorize the incidence and predictors of AKI after radical cystectomy. The results of the study highlight the importance of reporting such significant complication in a standardized fashion. In addition, it appears mandatory to counsel the patient preoperatively for the risk of development of AKI. Finally, we invite all authors to report their incidence and predictors of AKI and the further impact on CKD on the long-term. Nevertheless, this study has several limitations that might interfere with accurate data interpretation. The retrospective nature of the study prevented incorporation of various potentially modifiable risk factors in the analysis as smoking history, the rate of blood transfusion, operative time, the use of nephrotoxic drugs, perioperative medications and anesthesia management that had been shown to affect the occurrence of AKI after major surgeries (8, 16, 31). Furthermore, we admit the relative low number of AKI incidents that underpowered the statistical analysis; therefore, multi-institutional studies are highly recommended.

## CONCLUSIONS

We conclude that AKI is a significant morbidity after radical cystectomy that is associated with higher mortality rate. Patients with nephroureterectomy and those with high grade complications are more liable to develop AKI. Impact of AKI on the development of CKD on the long-term in this patient population is awaited. We believe this term is to be included during routine morbidity assessment of patients with radical cystectomy and urinary diversion.
